# Basal Cell Carcinoma and Hedgehog Pathway Inhibitors: Focus on Immune Response

**DOI:** 10.3389/fmed.2022.893063

**Published:** 2022-06-14

**Authors:** Donatella Gambini, Emanuela Passoni, Gianluca Nazzaro, Giada Beltramini, Gianluca Tomasello, Michele Ghidini, Elisabetta Kuhn, Ornella Garrone

**Affiliations:** ^1^Medical Oncology Unit, Fondazione IRCCS Ca' Granda Ospedale Maggiore Policlinico, Milan, Italy; ^2^Dermatology Unit, Fondazione IRCCS Ca' Granda Ospedale Maggiore Policlinico, Milan, Italy; ^3^Maxillofacial Surgery and Odontostomatology Unit, Fondazione IRCCS Ca' Granda, Ospedale Maggiore Policlinico di Milano, Milan, Italy; ^4^Department of Biomedical, Surgical, and Dental Sciences, Università degli Studi di Milano, Milan, Italy; ^5^Pathology Unit, Fondazione IRCCS Ca' Granda Ospedale Maggiore Policlinico, Milan, Italy

**Keywords:** Gorlin syndrome, Gorlin-Goltz syndrome, basal cell carcinoma, Hedgehog, *PTCH1*, Hedgehog inhibitors, immune response

## Abstract

Basal cell carcinoma (BCC) is the most common form of skin cancer, affecting more often elderly patients, but sometimes even younger ones, particularly if immunocompromised or genetically predisposed. Specifically, the Gorlin-Goltz syndrome, an autosomal dominant genodermatosis, also known as nevoid basal cell carcinoma syndrome, characterizes for multiple early onset BCCs. It is caused by a germline mutation in *PTCH1*, a tumor suppressor gene whose product is the key component of Hedgehog (Hh) signaling pathway, which also appears somatically mutated in more than 85% of sporadic BCCs. Hh pathway inhibitors vismodegib and sonidegib are currently indicated for BCC, in adults with advanced or recurred tumor following surgery or radiation therapy. The principal mechanism of action of these drugs is the inhibition of Smoothened (SMO), a transmembrane protein involved in Hh signal transduction, that plays a role in both cellular differentiation and cancer development. Some studies have reported effects of Hh pathway inhibitors at different levels of the immune response, from cytotoxic T cells to a modified local cytokines pattern. Given the specific relation between immune system and BCC development in some conditions, we will review BCC with focus on immune system changes mediated by Hh signaling pathway and induced by the inhibitors vismodegib and sonidegib in the treatment of BCC. Thus, we will give an overview of their effects on the local immune response, as well as a brief note on the supposed function of Hh pathway inhibition on the systemic one.

## Introduction

Basal cell carcinoma (BCC) is the most common form of skin cancer, with predominantly local malignancy. The high incidence especially in elderly patients, together with the increase of the global median age, make BCC a relevant public health issue representing about 80% of non-melanoma skin cancers (1).

Recently, new therapies have been approved for locally advanced disease, not suitable for surgical resection and/or radiotherapy, with good global results. Among these, there are vismodegib and sonidegib, two drugs that act inhibiting the Hedgehog (Hh) signaling pathway, one of the principal mechanisms involved in the complex molecular pathogenesis of this disease (2, 3).

Given the high incidence of BCC in immunocompromised patients, some studies were conducted to understand the local immune response elicited by the tumor, as well as the immunological modifications consequent to Hh pathway inhibition therapy.

In this review, we summarize the main features of BCC, its immunological findings, and the immune response to the treatment with the two approved Hh pathway inhibitors, vismodegib and sonidegib.

## Basal Cell Carcinoma

BCC originates from the basal cell layer of the epidermis, in which cells are constantly dividing to replace lost elements. Specifically, some studies aimed to determine the precise origin cell of BCC have identified a stem cell associated to hair follicle (4).

Many risk factors are well-known to promote BCC: UV light exposure, especially in low Fitzpatrick skin phototype people; personal history of skin cancer; older age; male sex; and exposure to certain chemicals (arsenic, coal tar, and other industrial compounds), photosensitizing drugs, or radiations (5–8).

Face, neck, and in general photo-exposed skin areas are the most involved sites of development of BCC, even if not exclusive.

Preferred therapies are surgical (classical excision, Mohs surgery, electrosurgery, cryosurgery, laser surgery), or locoregional treatment (radiation, photodynamic therapy, and topical drugs). When systemic therapy is required (for cases ineligible for local, surgical, or radiation therapy), Hh pathway inhibitors and the immunocheckpoint inhibitor cemiplimab are available and indicated (2, 3, 9).

## Pathology and Pathogenesis

Typically, BCCs are composed of small ovoid or round basaloid cells clustered in cords, trabeculae, islands, or nests, with peripheral palisading cells, and haphazard arrangement of centrally located cells. Intratumoral and stromal deposition of mucin, apoptotic cells and mitotic figures could be also observed, while perineural invasion is associated with a more aggressive disease (10, 11).

Based on the main growth pattern, different morphological subtypes have been described: nodular, superficial, cystic, infundibulocystic, pigmented, adenoid, fibroepitheliomatous, as well as the more aggressive basosquamous, micronodular, infiltrative, and sclerosing/morpheaform. Indeed, many cases share overlapping features of these different variants.

The molecular pathogenesis of BCC is complex and involves both sporadic somatic mutations and inherited genetic susceptibility (12–14). Sporadic mutations more frequently dysregulate Hh signaling pathway, an important pathway involved in many developmental and tissue homeostasis processes, affecting *PTCH1* gene in more than 85% of cases and SMO in about 10% of cases (15, 16). Some genodermatosis and other rare diseases include the development of multiple BCC among their phenotype. Among these, the most relevant for the high incidence of multiple BCC is the Gorlin-Goltz syndrome, a rare autosomal dominant genodermatosis caused by a germline mutation in *PTCH1* (17).

## Hedgehog Pathway

The pivotal role played by Hh pathway in basal cell proliferation, as well as its activation in BCCs, has been described since its discovery in 1980s (18, 19). Its biological relevance is further emphasized by the fact that it is phylogenetically well conserved in its entirety. This pathway appears essential for normal embryonic development, but in adults remains active for maintenance, renewal, and regeneration functions in the skin, hair follicles, and stem cells (20).

**Figure 1:**
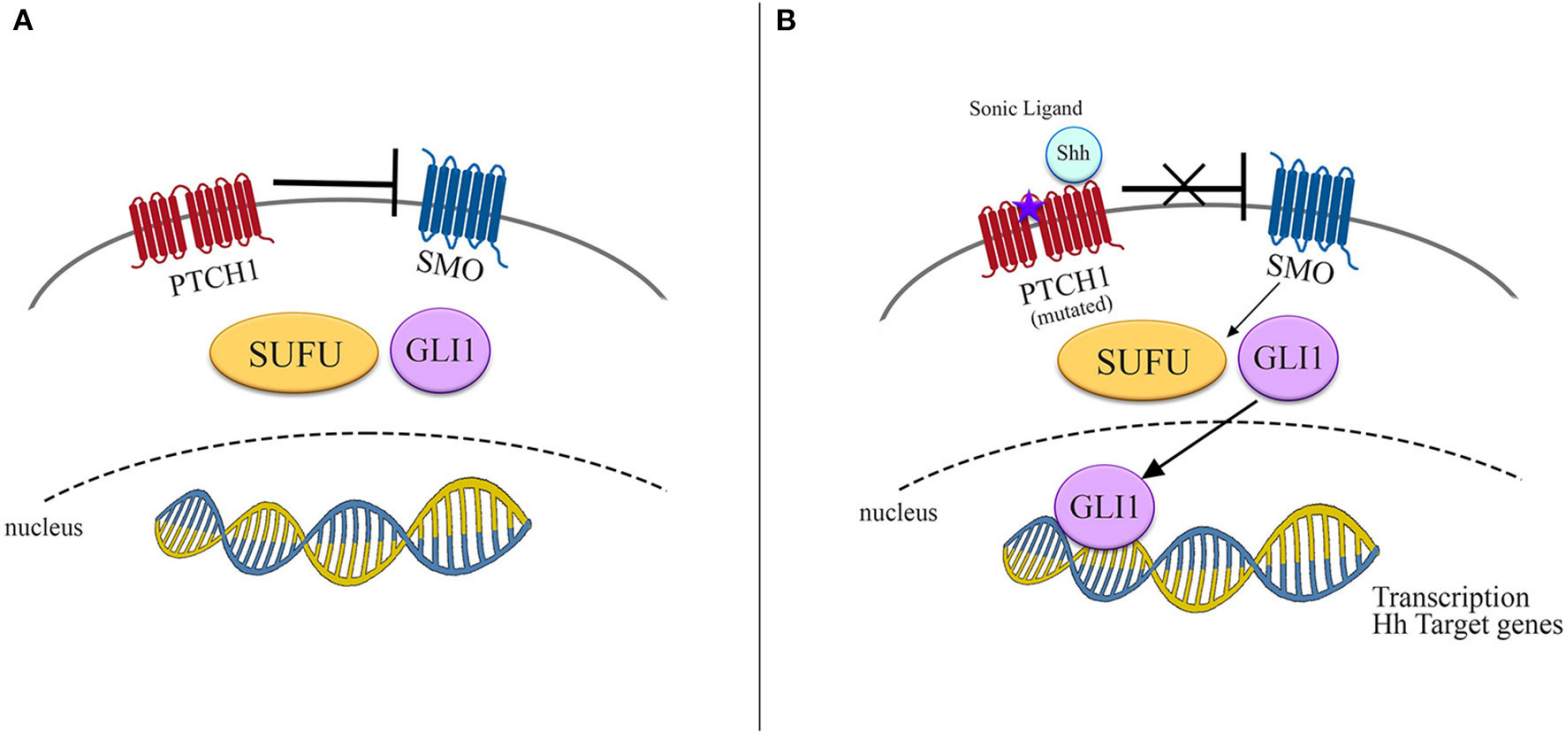
Schematic representation of Hedgehog signaling pathway. **(A)** In normal conditions SMO is inhibited by PTCH1 and no signals reach the nucleus to promote transcription. **(B)** In presence of either its ligand as Shh or mutated *PTCH1*, SMO is derepressed, promoting the transcription of Hh target genes by activation of SUFU/GLI1.

The protein PTCH1, encoded by *PTCH1* gene, acts as a canonical receptor for the Hh ligands Sonic (Shh), Indian (Ihh), and Desert (Dhh). The constitutively repressive function of PTCH1, present in normal conditions, is removed by the ligand-receptor binding, with a consequent derepression of the Smoothened (SMO) protein and its accumulation in cilia, in addition to phosphorylation of its cytoplasmic tail. SMO mediates a downstream signal transduction, including suppressor of fused homolog (SUFU), which lastly determines the activation of the glioma-associated oncogenes homologs (GLI) family of transcription factors (Figure 1) (21).

In addition to the abovementioned canonical activation of GLI through Hh-PTCH1-SMO pathway, mainly observed in normal tissues, an important role for a non-canonical and SMO-independent activation of GLI has been recently described in several types of cancer (22). Altered and uncontrolled activation of the Hh/GLI pathway is detrimental for the cell and has been shown to cause or facilitate the development of many neoplasms, representing the key molecular driver, especially in BCC and medulloblastoma (21).

## Gorlin-Goltz Syndrome

Gorlin-Goltz syndrome, eponymous for the nevoid basal cell carcinoma syndrome (NBCCS), is a rare genodermatosis, with an autosomal dominant transmission, due to *PTCH1* germline mutations. In rare cases, the development of Gorlin-Goltz syndrome has been linked to mutations either in *PTCH2* or *SUFU* (23). The disease is most commonly inherited with complete penetrance but variable expressivity. Approximately 70–80% of individuals with NBCCS have a family history, while about 20–30% results from a *de novo* pathogenetic variant (24). The estimated prevalence is about 1 in 60,000–164,000 people, with a predilection for Caucasians, and similar rates in both sexes (25).

The median age of clinical manifestations is 20 years, even if the onset can also be observed early in infancy. If BCC multiplicity and early onset represent the clinical hallmark feature of NBCCS, in many cases palmar and plantar pits, and abnormalities in different organs and apparatuses could be also observed in as many as 92% of patients. Skeletal (bifid or splayed ribs, cleft lip/palate, vertebral fusion, frontal bossing, pectus excavatum, syndactyly, hypoplastic thumbs, and the characteristic odontogenic keratocysts of the jaw), ophthalmologic (hypertelorism, congenital blindness, cataracts, strabismus, colobomas of the iris or retina), and neurologic (intellectual disability, seizures, and others) abnormalities are all criteria for the clinical diagnosis. Medulloblastoma could arise in childhood. Other rarer findings are cardiac and ovarian fibromas.

As abovementioned, when a loss-of-function mutation occurs in *PTCH1*, SMO becomes activated, upregulating the Hh signaling pathway, and allowing downstream activation of the zinc-finger transcription factors GLI1 and GLI2, the last mediators of this pathway (26, 27). The genetic mechanism called into question is the Knudson's two-hit model; when a pathogenetic mutation of one allele is inherited, in this case in *PTCH1*, a secondary acquired loss-of-function mutation could inactivate the other allele triggering tumorigenesis.

## Immunological Findings in BCC

The importance of the immune system in the carcinogenesis is well recognized for a long time, but more recently immunotherapy has started revolutionizing cancer therapy. The main interest in the field of tumor immunology has been focused on the tumor microenvironment (TME), where a local immune response to cancer cells could play a substantial role in both cancer development and treatment, as well as in prevention.

Cancer immunosurveillance is a highly dynamic process of interaction between cancer cells and the host immune system, entailing a balance between tumor-promoting and host-protective activities.

Some characteristics of BCC, especially its increased incidence in immunocompromised patients, have stimulated efforts and studies to clarify the complex mechanisms of local immune response, especially regarding TME. The local immune response was first assimilated to a delayed-type hypersensitivity with recognition of tumor antigens and a subsequent cell-mediated response. Such a response could be possibly reduced by suppressor cell activity that has been well documented in animal models with UV-induced skin cancers and also suggested in certain patients with multiple BCCs (28).

The most relevant immune cells in the TME are the so-called tumor-infiltrating lymphocytes (TILs), usually considered a favorable prognostic factor, because expression of the host immune response activation (29). Importantly, there are different subgroup populations of TILs, such as regulatory T-cells (T-regs), exerting an immunosuppressive activity, also mediated by secreted inhibitory cytokines, as IL10 and TGFβ.

For BCC, studies have revealed the presence of high concentration of T-regs, not only in TME, but also in the tumor bordering skin, chemoattracted by different chemokines, for example CC chemokine ligand 17, 18 and 21 (CCL17, CCL18 e CCL21). The T-regs in BCC TME account for about 45% of CD4+ cells (30). This fact is especially relevant considering the absence of T-regs in normal not UV-exposed skin (and in up to 20% normal human adult skin) and suggests a T-regs role also in normal skin surrounding BCC. TILs in BCC seem to be recruited from circulating cell pool rather than from resident T cells (31).

An elegant study has shown that Hh-driven BCCs exhibit an increase in myeloid-derived suppressor cells and a decrease in T cells, expression of an immunosuppressive TME. Specifically, Hh activation in keratinocytes through TGFβ signaling induction recruited bone marrow-derived cells, promoting tumor development in this mouse model. On the other hand, Tgfrb2 deficiency in bone marrow cells reduced tumor development (32).

The chemokine-mediated attraction of T-regs could also be mediated by the so-called cancer-associated fibroblasts (CAFs). Coherently, Omland et al. demonstrated a high presence of CAFs markers in BCC, while an intermediated type of fibroblast (between normal and cancer-associated type) was observed in the peritumoral skin, probably induced by UV stimulation (30).

UV exposure seems to be also a cause of both local and systemic immunosuppression, due to response to structural changes elicited in the chromophores, a variety of immune mediators are released, with a global immunosuppressive effect.

One of the most reliable hypotheses considers BCC an “immunogenic” tumor, albeit able to generate a protective niche in the TME. This “defective” anti-tumor response is more evident in immunocompromised people (i.e., transplant recipients, patients in therapy with immunosuppressive drugs, and HIV- infected patients) (30).

Moreover, a comprehensive analysis of the immune TME of BCC reported an attenuated state of the local immune system in BCC, revealed by a Th2 dominant TME (increased expression of both specific cytokines, such as IL-4, IL-10, and CCL2, and interferon-associated genes), as well as the presence of T-regs and immature dendritic cells (33).

Another evidence supporting a BCC-induced defective immune local response is the efficacy of therapies with a well know immunomediated mechanism, like both imiquimod and photodynamic therapy, in the treatment of BCC (34, 35).

## Hedgehog Pathway Inhibitors in BCC and Immune Response to Treatment

To date, two small molecules targeting SMO have been approved in the treatment of metastatic BCC or for patients with local advanced BCC ineligible for surgery or radiation therapy: vismodegib and sonidegib.

In 2012, vismodegib (Erivedge^®^) has been the first Hh pathway inhibitor approved by FDA for BCC. Structurally unrelated to cyclopamine, a natural alkaloid steroid and the first molecule known to act as a Hh pathway inhibitor (36), it binds with high affinity and specificity to SMO, producing a strong inhibitor effect on the Hh pathway (37).

**Figure 2:**
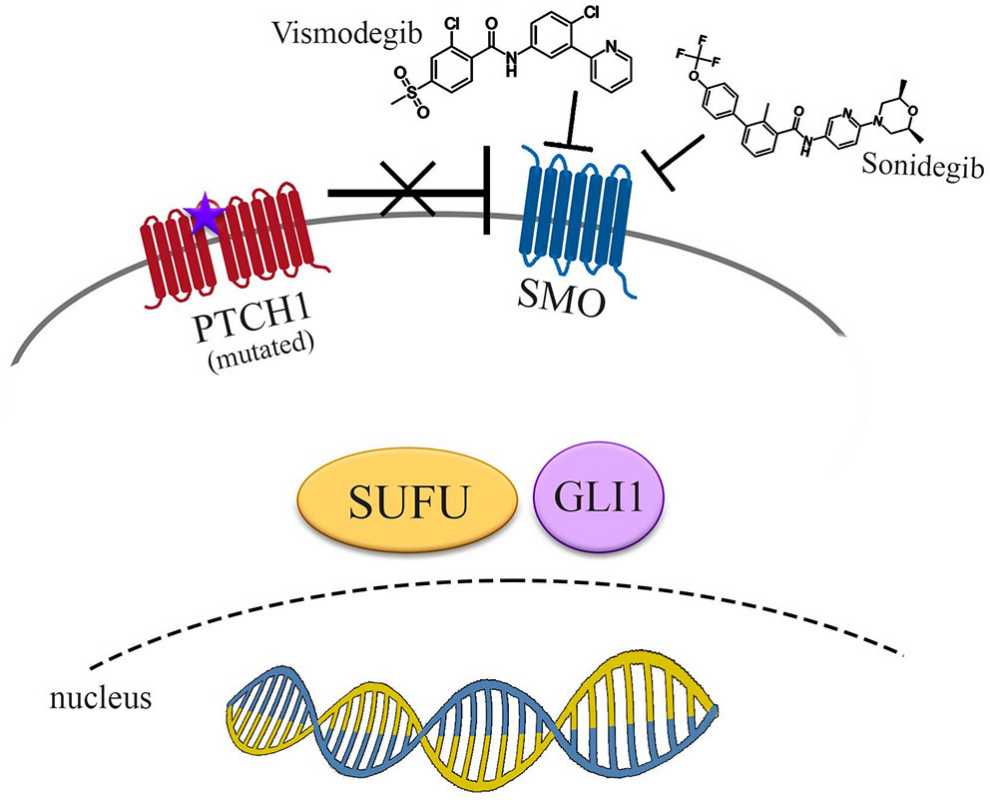
Pharmacological inhibition of Hedgehog signaling pathway. Vismodegib and sonidegib act inhibiting SMO, even if derepressed by mutated PTCH1. No signals reach the nucleus to promote transcriptional activity of Hh target genes.

Then, sonidegib (Odomzo^®^), which interacts with a SMO pocket preventing downstream activation of Hh pathway signaling, so exerting an antagonistic activity, was approved in 2015 (38) (Figure 2).

Four phase 2 studies (ERIVANCE, STIVIE and MIKIE for vismodegib, BOLT for sonidegib), were conducted using these two drugs. The reported efficacy outcome from the final analysis of ERIVANCE, STEVIE, and BOLT showed an overall response rate (ORR) up to about 70%, and always >50% in the local advanced setting. A lower ORR, as expected, was observed in the rarer metastatic disease, with ORR ranging from 8 to 49% (different percentages were reported with sonidegib at a dosage of 200 or 800 mg daily, and for both vismodegib and sonidegib either by investigator or central review) (39).

## Effects of Hedgehog Pathway Inhibitors on Local Immune Response

Many findings support the ability of Hh signaling to suppress the immune response against tumor (40). Using a genetical animal model of BCC, Grund-Groschke et al. showed an immunosuppressed TME in BCC when Hh signaling is activated. In fact, not only the accumulation of T-reg cells, but also an increased expression of immune checkpoint molecules including programmed death (PD)-1/PD-ligand 1 was observed (41).

Only one study focused on the effects of Hh pathway inhibitors on immune response specifically in BCC has been published (42). The authors analyzed biopsies from 23 patients with BCC, before and after 4 weeks of treatment with a Hh inhibitor (all but one with vismodegib, the remaining with sonidegib), moreover paired biopsies were available for 5 patients. They reported a significative change in the TME after therapy, characterized by the disruption of the immune privilege, and the promotion of a local adaptive immune response. In particular, in a tumor regression phase during therapy, they observed the upregulation of the MHC class I, and the increase of infiltrating immune cells (CD8+, CD4+, HLA-DR-class II+ mononuclear cells, CD68+ macrophages) at the cancer site (intralesional and surrounding). Moreover, they also detected an increased number of T-regs in both intra- and peritumoral site, even if the CD8+/Foxp3+ ratio was increased only in the intratumoral areas. Regarding cytokine and chemokine milieu, they observed in tissue biopsies an upregulation of genes coding for CCL18, CCL21, and CXCL9 after treatment. These chemokines, produced by both innate immune cells, like macrophages, and epithelial cells, have a role in the adaptive immune response and tumor suppression. Furthermore, the observed upregulation of vascular endothelial growth factor A during a response phase of treatment, supports the hypothesis of a granulation phase with angiogenesis in regressive lesions.

Another interesting observation is about T-cell receptor (TCR) and co-stimulatory ligands. When the Hh pathway is activated, the strength of the TCR signaling in mature peripheral T-cells is reduced, while T-cell activation is increased when Hh signaling is inhibited. This is very important about the potential direct effects of Hh pathway inhibitors on peripheral T-cells, and consequently, on the immune modulation and activation of an adaptive immune response (43).

Some reports indirectly contributed to support the role of Hh inhibitors in stimulating a local immune response. Particularly, Fosko et al. described a case of lichenoid dermatitis developed during a successful treatment of BCC with vismodegib. Of note, lichenoid dermatitis has been reported during immunosuppressive therapies, such as interferon and imiquimod, and the supposed involved mechanism implies at least in part a local immune response (44). Observing a very striking remission, authors supposed that vismodegib also acted by eliciting a local immune response, which could cause dermatitis.

Another probable mechanism invoked was apoptosis, but some evidences show a lack of apoptotic response *in vivo* during BCC treatment with vismodegib at conventional doses (45). Apoptosis has been instead observed with cyclopamine as well as with a new powerful Hh pathway inhibitor, capable of activating caspase 8-dependent apoptosis (46, 47).

A summary of local immunity modifications in both untreated BCC and during therapy with Hh pathway inhibitors is shown in Table 1.

**Table 1:** Local immunity modifications in untreated BCC and during therapy with Hh pathway inhibitors.

**Untreated BCC**			
**References**	**Intralesional/TME**	**Surrounding skin**	**Notes**
Omland et al. (30) and Omland (31)	Increased Tregs	Increased Tregs	Human BCC
	CAFs markers	Intermediated fibroblasts (CAF precursors?)	Human BCC
Kaporis et al. (32)	Th2 dominance (IL4, IL10, CCL2) Increased: - Tregs - Immature dendritic cells Unchanged: CD1b/c+ and CD11c+ Reduced: Langerhans cells (CD1a+)	Increased (slightly) T regs Unchanged CD1b/c+ and CD11c+ Not reduced Langerhans cells (CD1a+)	Human BCC
Grund-Gröschke et al. (40)	Increased Tregs	Not specified	Animal model
	Increased PD1- PDL1 expression		
**Treated BCC with Hh pathway inhibitors**			
**References (drug)**	**Intralesional/TME**	**Surrounding skin**	**Phase of treatment**
Otsuka et al. (42) - Sonidegib (22 pts) - Vismodegib (1 pt)	Upregulated: MHC I Genes for CCL18, CCL21, and CXCL9 Increased: - CD8+ - CD4+ - HLA-DR-class II+mononuclear cells - CD68+ macrophages - VEGF A - T cell -receptor signaling - CD8+/Foxp3+ratio	Increased: - CD8+ - CD4+ - HLA-DR-class II+ mononuclear cells - CD68+ macrophages Not increased: CD8+/Foxp3+ ratio	Regression
Fosko et al. (44) - Vismodegib	Lichenoid dermatitis (immunomediated?)	Not specified	Regression
Miller et al. (43) - Vismodegib	Lack of apoptosis	Not specified	Regression

*CAFs, cancer associated fibroblasts; TME, tumor microenvironment; VEGF, vascular endothelial growth factor*.

## Effects of Hedgehog Pathway Inhibitors on Systemic Immune Response

No data are available about the effects of Hh pathway inhibitors on the systemic immune response in patients treated for BCC. However, the effects of the systemic SMO inhibition on thymic and splenic T-cells have been studied in a mouse model. The results showed that the Hh signaling both promotes the γ-δ differentiation in the thymus and influences the T-cell pattern of distribution in the spleen. Consequently, the SMO inhibition causes the reduction of the γ-δ T-cells and γ-δ NKT cells both in the thymus and in the spleen, confirming a role for Hh pathway in the T-cell differentiation process (48).

*In vitro* studies demonstrated a significant role of Hh signaling in T-cell killing especially in controlling the cytotoxic T-cell functions and the degranulation process (49).

Some data are published on different local T-cell patterns during systemic pharmacological SMO inhibition. Yanez et al. reported a reduced inflammatory reaction in a mouse model of allergic airway disease. During systemic therapy, they observed not only a reduced percentage of basophils, eosinophils, mast cells and CD4+ T cell in the lungs, but also a decreased CD4+T cells infiltration in the mediastinal lymph nodes, as well as a reduced level of immunoglobulin E in the serum (48). Although not only local effects were observed, the systemic ones could be more likely correlated to the reduced local inflammation, so that the systemic effect should not be interpreted as primary, but as a consequence of SMO inhibition.

Interestingly, the effects of SMO inhibition on local immune system seem to be different in the skin as compared to the lung, because the Sonic Hh signal appears to have different downstream consequences in such tissues. Specifically, in the skin it promotes T-regs activity, favoring an immunosuppressive environment, while in the lung induces a Th2 response and so facilitating allergy/asthma (50–52).

## Conclusions

BCC is an extremely frequent skin cancer, in which the dysregulation of the local immune environment plays a remarkable role in the balancing immunosurveillance toward immune evasion. Hh pathway represents a well-known pivotal signaling system, upregulated in BCC. In the last decade, two drugs able to inhibit Hh signaling acting on SMO, vismodegib and sonidegib, were licensed for treatment of metastatic or locally advanced BCC, and local immune modifications during such a treatment start to be described. Studies focused on new SMO inhibitors and immunocheckpoint inhibitors are ongoing, in order to verify a greater efficacy and maybe to overcome resistance. Further investigations are needed to evaluate the possible impact of SMO inhibitors on the systemic immune response, as well as the potential effect of such drugs in the treatment of other neoplasms.

## Author Contributions

DG: conceptualization, writing, editing, revision, and figure preparation. EK: conceptualization, editing, and revision. EP, GN, GB, GT, MG, and OG: conceptualization and revision. All authors contributed to the article and approved the submitted version.

## Funding

This study was funded by Grant Ricerca Corrente 2021, Italian Ministry of Health.

## Conflict of Interest

The authors declare that the research was conducted in the absence of any commercial or financial relationships that could be construed as a potential conflict of interest.

## Publisher's Note

All claims expressed in this article are solely those of the authors and do not necessarily represent those of their affiliated organizations, or those of the publisher, the editors and the reviewers. Any product that may be evaluated in this article, or claim that may be made by its manufacturer, is not guaranteed or endorsed by the publisher.
